# What do Professional Helpers Describe as Important Competence when Supporting Persons Bereaved by a Drug-Related Death

**DOI:** 10.1177/00302228241265150

**Published:** 2024-07-22

**Authors:** Torill Hauge Totland, Kristine Berg Titlestad, Sari Kaarina Lindeman

**Affiliations:** 1 377787 Haugesund Municipality, Haugesund, Norway; 2 366044 Western Norway University of Applied Sciences, Bergen, Norway

**Keywords:** drug-related death, bereavement, grief support, professional competence, caregivers

## Abstract

Bereaved persons following a drug-related death (DRD) experience significant stress, an increased risk of health-related problems, complicated grief reactions and a risk of higher mortality. Despite the support received from professional helpers being important, research has yet to examine their perspectives to understand and meet the bereaved’s needs and increase the helpers’ competence. Hence, this qualitative study explored professional helpers’ perspectives on essential skills and competencies when supporting bereaved following DRD. We conducted focus group interviews with 29 professional helpers from diverse health and welfare services across six Norwegian municipalities. Reflexive thematic analysis generated three main themes: *Diversity of competence, Basic human competence* and *Balancing act between different considerations.* It is crucial that municipal services explicitly define the needs of bereaved persons following a DRD and determine the necessary professional competencies and appropriate intervention levels required.

## Introduction

There has been little focus on bereaved persons following a drug-related death (DRD) in research worldwide, although the field has been given more attention in recent years. Research has already shown that bereaved persons following a DRD are likely to experience increased stress levels, complicated grief reactions, negative health outcomes, and an increased risk of mortality (Christiansen et al., 2020; Titlestad & Dyregrov, 2022; Titlestad et al., 2021a). Their unique grief responses may include ambivalent feelings of shock, relief, anger, shame and guilt, potentially leading to an emotional overload. They may also struggle with relational issues and social isolation (Lambert et al., 2021; Titlestad et al., 2021a; Titlestad et al., 2021b). Bereaved persons with substance use problems may face a higher risk of experiencing complicated grief reactions (Masferrer et al., 2017; Selseng et al., 2023).

Many bereaved persons after a DRD have experienced challenging encounters with support services, including being met with prejudice and stigmatic attitudes, and a lack of kindness and understanding (Templeton et al., 2016; Titlestad et al., 2021a; Valentine et al., 2018). However, little research has been conducted on the perspective of professional helpers and their descriptions of encounters with bereaved persons after DRDs. A recent systematic review (Reime et al., 2022) concluded that there had been no studies on professional helpers’ follow-up of bereaved persons after a DRD based on the helper’s perspective. Reime and co-researchers argued that more knowledge was needed, both for educational purposes and to improve the quality of health and welfare services based on the needs of the bereaved. Two Norwegian studies which examined professional helpers in public health and welfare services and their contact with bereaved persons during the acute phase following a DRD, have confirmed the need for more knowledge about helpers’ perspectives (Løseth et al., 2022; 2023).

In this project we have relied on the definition of DRDs provided by the Norwegian Directorate of Health (2014a). Drug related deaths are deaths caused by the direct intake of narcotic substances, and by more indirect causes such as accidents, violence, infections and other health issues related to the use of drugs (Norwegian Directorate of Health, 2014a). In 2021 106,699 persons died due to drug overdose in the USA (32.4 per 100,000), and in Europe, the number of drug-induced deaths was estimated to be 6677 persons (Centers for Disease Control and Prevention, 2023; European Monitoring Centre for Drugs and Drug Addiction, 2023). For each DRD there is an estimate of at least ten bereaved persons (Møgster et al., 2018). Given the many bereaved persons, research that may improve the quality and scope of support provided to this group can have significant implications for public health (Dyregrov et al., 2020).

As with other qualitative studies, it is essential to present the results within the context in which the study was conducted. Thus, descriptions of the Norwegian welfare state must be emphasized as part of this context. Norway is a welfare state that gives bereaved persons following a sudden death the right to receive assistance and support from public health services (Dyregrov & Kristensen, 2021). Norwegian health care guidelines require services to be delivered responsibly, ensuring providers have adequate professional competence or refer patients when necessary. Emphasis is placed on the need for collaboration between various health and care services, with municipalities taking on greater responsibility for resident care (Health and Care Services Act, 2011, section 4-1; Report No. 47 to the Storting (2008–2009); The Health Personnel Act, 1999, section 4; Willumsen, 2016). Municipalities must utilize expertise across professional disciplines and services to ensure coordinated services and professional breadth in the care provided to individuals with significant and complex needs (Norwegian Directorate of Health, 2019).

The inclusion of the diagnosis of Prolonged Grief Disorder (PGD) in the World Health Organization’s diagnostic classification system, ICD-11 (cf. WHO, 2023), has led to an awareness of grief as a diagnosis and the follow-up care for bereaved individuals internationally (Killikelly et al., 2021). The Bereavement Network Europe (BNE) is working towards establishing a standardised model for effective grief treatment and support to address the limited understanding of diagnosis, assessment and care for bereaved individuals. The BNE seeks to gather expert recommendations on determining the appropriate levels of intervention and the essential components involved in assisting bereaved individuals (Killikelly et al., 2021). The BNE suggests a treatment approach presented as a three-tiered model with different levels of intervention for the follow-up of bereaved persons. This model is known and used in health-related work in several countries (Killikelly et al., 2021) and is based on the public health model for psychosocial follow-up of bereaved individuals (cf. Kristensen, 2021). The three-tiered model for treating bereaved individuals offers various levels of intervention for ongoing support. This model suggests that support can be provided at different tiers, ranging from universal assistance to more specific and targeted aid. It is important to recognize that the level of support required varies for each individual, with sources of help ranging from family and friends at the universal level to professionals trained in grief and trauma treatment at the indicated level (Killikelly et al., 2021).

Experience with lack of support, public stigma, self-inflicted stigma and complicated feelings towards support services may lead bereaved persons after a DRD to avoid asking for or accepting the help they need (Dyregrov & Selseng, 2022; Templeton et al., 2017). Bereaved persons who themselves use drugs have expressed the need for competent help with both their grief and their drug-related problems, which calls for professional helpers to have an understanding and knowledge of their unique situation and issues (Selseng et al., 2023).

In a recent study conducted by Løseth and colleagues (2022), it was found that professional helpers’ attitudes and past experiences with clients’ use of illegal drugs can affect their evaluation of the support needs of bereaved individuals during the acute phase following a DRD. It is, therefore, important for professional helpers to gain sufficient knowledge and competence to understand the reactions of bereaved people following DRDs and how to get into a position to help them (Løseth et al., 2022; 2023).

Professionals need different types of competence to perform their tasks and responsibilities. Comprehensive professional competence may include theoretical knowledge, occupation-specific skills and personal competence (Skau, 2017). When working with people, the competence of moral actions and judgment, so-called phronetic competence, is also considered significant (Øverlid, 2009). Relational dialectics recognize our existential, fundamental and shared terms as human beings. People are individuals with their understanding of their experiences and, simultaneously, part of a community. In a professional relationship, the practitioner must be able to immerse themselves in the client’s experiential world while also maintaining an observing and reflective stance concerning the other person and the dynamics within the relationship. Thus, self-reflexivity and self-delineation become essential concepts (Schibbye, 2009). Recognition is significant in relational dialectics, and includes an attitude of listening, understanding, sharing emotions and having a fundamental respect for the other’s experience. Mutual recognition involves seeing a situation from one’s own perspective, switching to the other’s perspective, and then returning to one’s own viewpoint (Aamodt, 2003; Schibbye, 2004). In light of relational dialectics, professional practitioners must be compassionate, show empathy, hold awareness of their feelings, and be present as human beings. However, this must be done attuned, ensuring that their emotions do not dominate or become invasive toward the client (Aamodt, 2003; Schibbye, 2009).

How bereaved people following a DRD are approached and supported may affect their health. Bereaved persons who rate received professional help as highly satisfactory have a better social health score than bereaved persons who rate professional help as poor. Reduced social health is strongly associated with withdrawals from others and the perception that others have withdrawn from them (Kalsås et al., 2022). Therefore, gaining a deeper understanding of helpers’ perspectives can potentially shape and improve public health and welfare services practices for bereaved persons. Hence, the aim of this study is to explore what professional helpers from municipal health and welfare services consider to be important competence when supporting bereaved persons following a DRD. It will contribute to a greater understanding of a perspective that has been marginally explored so far.

## Methodology

This study was based on the data from the project Drug Death Related Bereavement and Recovery (the END project in Norwegian). The END project is a major Norwegian project that studies bereavement following a DRD, and the help given through health and welfare systems (Western Norway University of Applied Sciences, 2023).

Data for this study was collected through focus group interviews and analysed by reflexive thematic analysis (cf. Braun & Clarke, 2022). The study is qualitative with an exploratory, inductive approach.

### Recruitment

Recruitment for the project was conducted in the spring of 2019. Crisis teams in municipalities that had participated in the Norwegian Directorate of Health’s pilot project for the prevention of overdoses were asked to establish contact with helpers who had had encounters with bereaved persons following a DRD through their work. Helpers who wished to participate in the study were informed that participation was voluntary, and that anonymity would be ensured. All participants signed informed consent forms.

A total of 105 helpers from six different municipalities were included to the part-study of helpers in the END project, organized into four different groups: (1) First-responders and acute support personnel; (2) Representatives from different non-governmental organisations; (3) Professional helpers from municipal services who meet with bereaved persons; and (4) Leaders of different municipal health and welfare services. A total of 24 focus group interviews were conducted in this part of the project, one for every group in all six municipalities.

### Sample

To address the research question of this study, data from the six focus group interviews with professional municipal helpers (Group 3), was used. The sample consisted of 29 professional helpers, eight men and 21 women. The participants had different professional backgrounds, including social workers, nurses, physicians, social educators, public health nurses, psychologists and child welfare officers. Municipalities represented in the focus group interviews differed in terms of population and structure, and they were both urban and rural.

### Focus Group Interviews with a Theme Guide

Data were collected through semi-structured focus group interviews. The applied theme guide focused on helpers’ experiences, roles and tasks in their contact with bereaved persons following a DRD, as well as their collaboration with others and any needs the helpers might have.

Two researchers from the project group conducted the interviews during autumn 2019 and spring 2020. Group sizes varied from two to seven people. The interviews, lasting 1.5–2.5 hours were recorded and later transcribed by a professional transcriber. The six interviews provided a total of 144 pages of transcribed text.

### Analysis

Data was analysed using reflexive thematic analysis as described by Braun and Clarke (2006; 2019; 2022). This analysis consists of six steps: familiarization with the data, coding the data, generating themes, reviewing potential themes, defining and naming themes and producing the report. The process is dynamic, and the researcher can move back and forth between the steps of the analysis (Braun & Clarke, 2006; 2022).

The reflexive emphasis requires the researcher to be clear and transparent about their own contribution to the analysis and theme generation (Braun & Clarke, 2006; 2019; 2022). The first author of the article is a health professional, with occupational experience from municipal health and social welfare services. The second author is a co-leader of the END project. The third author is a researcher in the END project and has a lot of experience as a professional helper in municipal substance use services. The authors have discussed throughout the analysis how our professional and personal experiences and competence have impacted our analytic process.

During the first step of the analysis, the data material was read and listened to several times. Data from all six interviews was retained, based on the desire and intent to gain a broad understanding and analysis of the data in order to explore the research question in a field that has thus far been insufficiently studied. In step two initial codes were developed. During steps three to five of the analysis, themes and subthemes were generated through a process of sorting codes, categorising, defining and naming themes and making adjustments. The authors had frequent discussions on how to understand data and define themes in this part of the analysis. The authors’ different occupational backgrounds added breadth to these discussions. The sixth step of the analysis is writing the manuscript. A thorough analysis and work process resulted in three main themes, each with three subthemes.

### Ethical Considerations

This study was approved by the Norwegian Agency for Shared Services in Education and Research, ref. no. 525,501. Transcribed material and audio files were securely stored on the researcher server at Western Norway University of Applied Sciences. Data and results have been processed and presented in a manner that ensures the anonymity of the participants.

## Results

**Figure 1.**
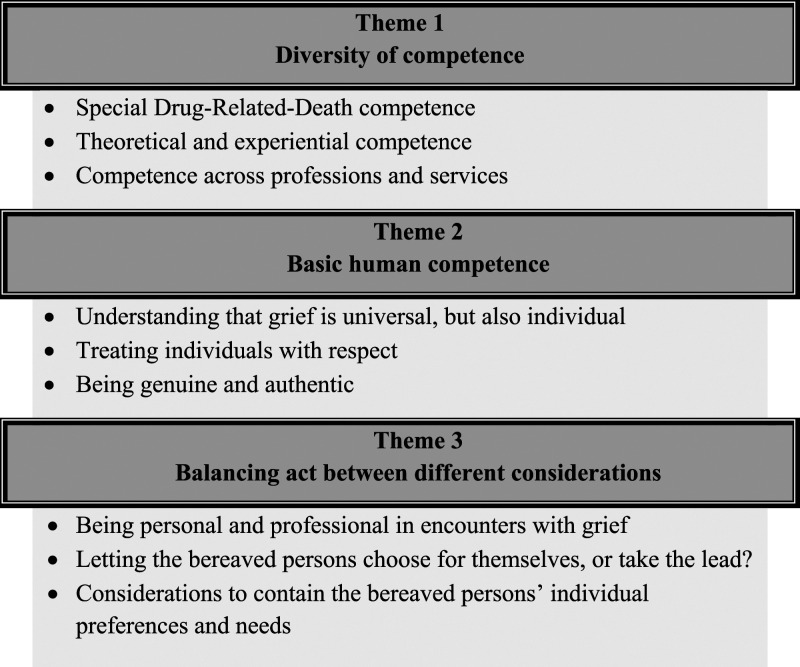
Overview of themes and subthemes.

Three themes reflect the professional helpers’ descriptions of what they believe to be important competence when supporting bereaved persons following a DRD: (1) Diversity of competence, (2) Basic human competence, and that helping requires a (3) Balancing act between different considerations. For each main theme, three subthemes were defined (Figure 1). Participants and interviews were assigned codes and are referred to as follows: 17/4 (participant no. 17, interview no. 4).

### Theme 1: Diversity of Competence

This theme reflects that the unique and complex needs of bereaved persons require broad and diverse competence from support services. Three subthemes were generated: (1) Special DRD competence; (2) Theoretical and experiential competence and; (3) Competence across professions and services.

#### Special DRD Competence

The professional helpers considered it as important to understand and know how to reach and help bereaved persons after a DRD in their unique situation. They described an understanding of the comprehensive burden on the bereaved prior to and following the death, and that their grief and emotional reactions can be complex. Conflicts, broken relations and the significant effect of stigma were considered part of this burden. Furthermore, it was brought out that several practical and legal issues had to be managed before grief could be addressed.

Some of the helpers described that bereaved persons following a DRD were living in an “extreme normal” situation, which is important to be aware of:

(...) then there’s the normal in the abnormal, or the abnormal in the normal, it becomes, in a way, normal. (...) they’ve started thinking these strange, extremely strange thoughts that other people who are on the outside may react to (...) And that’s really scary, I think. I think we need to keep this in mind. (14/3)

To understand the situation of bereaved persons who use drugs, the experiential competence of helpers from substance use services was considered especially valuable. These helpers, drawing on their knowledge of the deceased, the local network of people using narcotics, and problematic drug use in general, provided expertise on how to reach bereaved individuals who use drugs and what support these individuals would need to manage the situation. The helpers expressed concerns about the health-related burdens that bereaved persons who use drugs might have following a death, including fear of their own death, guilt and increased substance use. Helpers in substance use services described that they, based on this knowledge, were focused on not leaving bereaved persons alone after a death, and that they actively sought out those who withdrew from others. Establishing a sense of community, making room for listening and daring to ask difficult questions were all factors that helpers described as important. Based on their experience, the helpers also acknowledged that services to bereaved persons using drugs had to maintain a low threshold and a high level of accessibility for them to manage to accept the help offered.

#### Theoretical and Experiential Competence

This subtheme contains that helpers described both specific theoretical, professional knowledge and occupational experiential competence to be important. Theoretical knowledge of grief, trauma and crises was appraised as relevant. Many helpers viewed occupational experience gained from various encounters with people as key competence, partly to feel confident in their role as a professional helper. A sense of personal, professional confidence was regarded as important when supporting bereaved persons in such a unique and demanding life situation. One helper described this as follows:

(…) how one should behave comes with experience, so I might have behaved much differently, (…) been more secure in what I should do and had to do and, well, for the next of kin now than what I did eight–nine–ten years ago (…). So there is something about that confidence. (28/6)

Although most helpers expressed that they had some knowledge of various forms of grief, trauma and crises, several called for increased theoretical knowledge in this field.

#### Competence Across Professions and Services

The helpers described that supporting the bereaved after a DRD was complex work, and that collaboration and mutual exchange of knowledge across disciplines and professions would be beneficial. Helpers in mental health services expressed that the knowledge professionals in substance use services possess is particularly important.

Despite the wide range of professional and occupational competence in municipal services, referral to specialist services was considered necessary for some bereaved persons after DRDs. This called for helpers to make professional assessments of what can be considered normal reactions to grief and what might require more specialized follow-up. Some of the helpers felt confident in such assessments, while others wanted more knowledge and experience to determine when a referral was necessary. Collaboration with general practitioners was essential when sending such referrals.

Some of the helpers argued that the follow-up of bereaved persons should be provided by specialist services. Others expressed that specialists were not necessary, but that the bereaved needed someone professional, regardless of service level. Some of the helpers also pointed out that peer involvement, social networks and services from various religious and life philosophy communities might provide valuable competence and support for bereaved persons following a DRD.

### Theme 2: Basic Human Competence

This theme illustrates how helpers emphasized the significance of basic, universal human needs to be seen, heard and understood when supporting bereaved persons after DRD. The following three subthemes were developed: (1) Understanding that grief is universal, but also individual; (2) Treating individuals with respect and (3) Being genuine and authentic.

#### Understanding that Grief is Universal, but Also Individual

This subtheme reflects how helpers described and validated the grief and grief reactions of the bereaved persons, and how they showed an understanding of their individual needs. Helpers acknowledged that grief and crisis reactions affect all of us, as human beings. This may seem obvious, but the helpers noted that bereaved persons living lives dominated by drug use live in an “extreme normal” situation, and that many of these persons have experienced countless losses. Several of the helpers expressed concerns that this burden was not taken seriously enough, either by the bereaved persons themselves or by support services. Due to this, helpers described how they often have had to normalize and validate common grief reactions. Helpers described that many bereaved persons using drugs have not received, been able to accept, or understood their own need for help to process their losses.

Helpers explained that the situation for bereaved families also could be characterised by a stressful life situation over time, and by stigma and complicated emotions, such as guilt, shame and fear. Helpers described how it was important to confirm, validate and cautiously address a range of emotions and grief reactions. The needs of bereaved persons after DRDs were viewed as complex and individual, and the helpers emphasized that it consequently was necessary to differentiate the assistance given.

A few of the helpers argued that since grief is common, psychoeducation and self-help strategies could be useful and sometimes sufficient. These helpers also argued that social networks and society at large should play a role and have a responsibility to help bereaved persons cope with their grief. From this perspective, public information, openness about DRDs and reducing stigma were all considered as important factors.

#### Treating Individuals with Respect

This subtheme involves respecting the individual and acknowledging each person’s unique perspective. Respect was reflected through an emphasis on supporting the bereaved individuals from their unique point of view, a sensitive approach that could offer an opportunity to provide further assistance. Helpers emphasized offering, establishing, and maintaining relationships with the bereaved persons to support them in their needs. Several noted that helping children required particular respect and more specialized competence.

Many described an active approach to construct a broad and respectful narrative of the deceased, by talking about the person behind the problematic substance use, and by being open to listening to diverse stories from the bereaved friends and family. Helpers also stated that they arranged a worthy memorial service, with white tablecloths and candles. Through their words and actions helpers focused on showing respect for the bereaved and the deceased.

#### Being Genuine and Authentic

This subtheme reflects how helpers used their personal competence and experience, and what it is like to be a human being when coping with loss, grief and strong emotions. Helpers described how they used the breadth of their relationship skills and professional and human experience in their contact with bereaved persons. Being close, including at an emotional level, was emphasized and acknowledged as important and essential in this contact.

Some of the helpers had experienced complicated emotions and thoughts themselves after a DRD, for instance wondering if they should have done more for the deceased. Several described how it was significant to get emotionally close to people on a human level, but that it was burdensome to contain painful stories over time. It was noted that if this stress on helpers was not addressed, it could become an obstacle when supporting the bereaved.

### Theme 3: A Balancing Act Between Different Considerations

This subtheme reflects the helpers’ descriptions of having to make assessments that involve weighing different considerations and perspectives. Three subthemes were generated: (1) Being personal and professional in encounters with grief; (2) Letting the bereaved persons choose for themselves, or take the lead?; and (3) Considerations to contain the bereaved persons’ individual preferences and needs.

#### Being Personal and Professional in Encounters with Grief

This subtheme involves the balance of being close to the bereaved and managing one’s own, personal feelings, while at the same time keeping enough distance to ensure a professional functioning. Some of the helpers’ descriptions of their encounters with bereaved persons showed that they were emotionally close, but that they also needed to be able to withstand and accommodate strong emotional expressions of others, such as anger, insults and accusations in order to obtain power of action. The helpers viewed such situations as expressions of the bereaved person’s despair and grief, and helpers realised that they had to take a metaperspective and try to sustain a more distanced view of the situation. To manage this balancing act, knowledge of and experience with grief was helpful, as this avoided them from taking strong emotional expressions personally, and helped them tolerate them. One helper described this as follows:

(…) being able to accommodate the grief and not take things personally if there are, I mean, the different expressions of grief, and that you simply become a little more prepared for it (…). There are many times when we’re the ones who bear the brunt of it, when they’re in shock and grief. I’ve been scolded so many times for killing their child, that I didn’t look after them well enough. You just have to take it. (16/4)

Mastering the balance between acknowledging and managing one’s own feelings, understanding and tolerating the emotional expressions of the bereaved persons and assisting them with their practical and emotional needs calls for a broad range of competencies from helpers, including personal competence and professional self-confidence.

#### Letting the Bereaved Choose for Themselves, or Take the Lead?

This subtheme involves the balancing act of letting the bereaved persons choose for themselves whether they want to accept help, or whether helpers should take a more leading role in getting them to accept. Helpers wanted to respect the autonomy of the bereaved, while at the same time, they were aware that bereaved persons might have needs they are unable to manage on their own. Several of the helpers described knowledge of what bereaved persons might need, and that they had methods of getting into a position to offer help, including creating future opportunities for contact by asking for permission to call back after a while. The helpers reflected on whether being proactive and assertive in contact with the bereaved persons might be crossing a line, or whether it is the right thing to do.

#### Considerations to Contain the Bereaved Persons’ Individual Preferences and Needs

This subtheme includes how helpers try to attend to and respect the wishes and needs of the different bereaved persons. Helpers had experienced trade-offs in order to protect and respect the various unique perspectives of the bereaved individuals, as their interests may be contradictory or even conflicting.

Helpers naturally regarded the deceased’s friends as bereaved persons, albeit with fewer formal rights than family members. The deceased may have explicitly expressed a wish to refrain from contact with their family while they were alive, but their bereaved family members may still have formal rights to reach information after their death. Bereaved friends and family members might have different knowledge of the deceased’s life, and also of the circumstances of the death. It was considered challenging to determine what could be told to whom, in light of the duty of confidentiality. Legal implications were thus included in the helper’s considerations. One way of dealing with protecting different perspectives and legal considerations was to find a balance of listening and talking, with particular emphasis on listening when professional helpers felt bound by their duty of confidentiality.

Many of the helpers described how they adapted their actions to the wishes and perspectives of the bereaved persons. One recurring example was the issue of whether they themselves, along with friends who live lives dominated by substance use, should attend the funeral. Helpers stated that they did not attend if the bereaved family did not welcome their participation. To meet the bereaved friends’ need for rituals and gatherings, helpers then often organized their own memorial services. In these situations, helpers had to accommodate both the friends’ grief and the disappointment of not being permitted to attend the funeral.

To sum up, helpers described how they tried to provide the different bereaved persons with the information and support they wanted and were entitled to, but that this required considerations of ethical, moral and legal nature.

## Discussion

Each of the themes from the results reflects competencies the helpers described as important when supporting bereaved after DRDs: diversity of competence, basic human competence, and that helping requires a balancing act between different considerations. The results will now be discussed under three headings, corresponding to these themes.

### Diversity of Competence

The grief experienced by persons bereaved by DRDs has previously been described as complex and unique (da Silva, 2007; Dyregrov et al., 2020; Titlestad et al., 2021b). This group of bereaved persons may have been in a difficult situation for a long time, and the stigma associated with drug-related life and death adds additional burdens. Research indicates that the significant burdens can lead to health issues and even premature death among the bereaved (Christiansen et al., 2020). Being professional involves performing a task with the necessary qualifications and suitability (Skau, 2017). Competent help delivered with sensitivity, respect for the individual and with kindness can significantly impact the perceived quality of help, reduce feelings of stigma, and have a positive health effect on the recipient (Kalsås et al., 2022; Walter et al., 2017). Therefore, the competence possessed by professional helpers is highly significant. Qualitative studies conducted by Løseth et al. (2022; 2023) indicate that professional helpers during the acute phase following a DRD often have insufficient knowledge regarding the unique circumstances experienced by the bereaved. This suggests that it is crucial for professional helpers to acquire the necessary knowledge and competence to comprehend the reactions of the bereaved in order to effectively assist them (Løseth et al., 2022; 2023). However, the long-term helpers involved in our present study described that they possessed both knowledge and understanding of the bereaved individuals’ situations. They acknowledged that there is something distinct about experiencing bereavement following a DRD, and their descriptions indicate that follow-up of bereaved persons requires a special understanding and approach. The distinctiveness was described by the burden the bereaved persons may have carried over time, both prior to and following the death, such as conflicts, broken relations, practical issues, complex emotions and stigma.

Both public stigma and self-inflicted stigma may influence whether and how the bereaved will receive help, and whether they are able to accept the help offered (Dyregrov & Selseng, 2022; Templeton et al., 2017; Titlestad et al., 2021a). Participants in our study did not express any stigmatizing attitudes towards the bereaved persons or the deceased. However, some helpers expressed that they had felt insecure in certain situations, for example due to fear of rejection or strong emotional reactions from the bereaved persons. This may be an important perspective as the bereaved may be particularly sensitive to what helpers say and do, given their experience with stigma. They may interpret helpers’ expressions as stigmatizing or unfriendly even when they are not (cf. Titlestad et al., 2021b; Valentine et al., 2018). Potential anticipated stigmatization by the bereaved, combined with uncertainty on the part of helpers might result in difficult interactions between them, and in the worst case lead the bereaved persons to feel stigmatized or neglected. From the perspective of professionals, understanding this may be key to getting into a position to help bereaved persons who withdraw or do not feel worthy of help, as well as those who express strong emotions. Research shows that bereaved persons in difficult life situations need support from helpers who are confident and secure in their professional roles (cf. Thuen & Dyregrov, 2016). The helpers in our study described that it is professionally challenging to obtain a position to help bereaved persons following a DRD and to understand and manage their strong emotional reactions. The results show that helpers require theoretical and experiential knowledge to handle the complexity and diversity of the bereaved people’s needs. Professional experiential competence appears particularly important in maintaining the confidence of helpers in challenging situations. Further, ensuring theoretical competence regarding grief, grief reactions and the unique grief associated with drug-related death is of great essence.

The helpers described how it can be incredibly demanding to assist bereaved persons using drugs in their grief. This is both due to multiple, concurrent life challenges, and because the bereaved person’s coping strategies may involve increased drug use and social withdrawal. This perspective is supported by Selseng et al. (2023), as bereaved persons using drugs describe how important it is for them that their unique life situation is understood holistically, that they receive help for both their grief and substance use issues, and that the relationship between their substance use and grief is understood by professional services. This bereaved group’s need for complex and competent help can be particularly challenging, and clearly defining the appropriate level of competence and intervention seems essential.

In many countries, including Norway, municipalities have a clear responsibility to provide support for residents dealing with major and complex difficulties. Professionals from various sectors must collaborate and leverage their collective expertise to meet the diverse needs of these individuals (cf. Norwegian Directorate of Health, 2019). Mental health and substance use services call for a multidisciplinary approach, requiring vast expertise due to the broad variation of causes, challenges and needs (Norwegian Directorate of Health, 2014b). In WHO’s Comprehensive Mental Health Action Plan 2013–2020, a multisectorial approach is emphasized to develop good quality mental health services. The action plan calls for a collaboration between professional services and more “informal” mental health providers such as family and non-governmental organizations. It also urges specialized health workers to supervise others who provide mental health support (WHO, 2013). The professional helpers of this study described the need for competence across professions, services and service levels as essential when providing support for bereaved persons following a DRD. The bereaved persons may, according to helpers, need easily accessible low-threshold services. However, some will require follow-up at a specialist level. Several municipal helpers expressed a wish for more competence, both to provide support to bereaved persons who seek their help, and to assess and determine when the bereaved people’s grief requires more specialized care. This provides arguments for an intentional interdisciplinary collaboration in municipal services, and leads to questions regarding who and what level of service should support bereaved persons following a DRD. Prolonged grief disorder is now a medical classification in the ICD-11 diagnostic system (WHO, 2023), although not all bereaved persons will have symptoms that meet the criteria. The bereaved may nevertheless need systematic and competent care related to their unique grief, many for an extended period (Titlestad & Dyregrov, 2022). In Europe, the Bereavement Network Europe has been established with the aim to develop guidelines on how to assess and treat PGD and bereavement, and for clinicians and researchers to share knowledge on this field . The BNE suggests a three-tiered approach to bereavement care, ranging from support provided by non-professionals at the general level, to care and treatment provided by professional specialists at the indicated level (Killikelly, 2021).

There are aspects of a DRD and the consequences for the bereaved persons that may require specific expertise and knowledge at a group level. At the same time, one of the needs that appears to be of particular importance is the ability to understand the bereaved persons individually and with sensitivity, which places great demands on those who provide and organize support services for bereaved persons following a DRD. How we understand and explain drug use is significant, as it affects how we approach bereaved persons, but also how they understand their own history (Meen et al., 2024). As emphasized by Templeton et al. (2016) in previous research it is important to be aware that each bereaved person experiences their situation and the help they receive differently, which implies the necessity of individually adapted assistance. This perspective was confirmed by the descriptions made by this study’s participants of how they as helpers had to support the bereaved in a particularly sensitive and individual manner to be able to help.

It appears essential that the bereaved following DRDs are actively defined and understood as a group of bereaved with unique and complex, yet individual needs. Then attention can be brought to which competencies helpers need in order to provide appropriate and competent assistance and how interprofessional competence can be utilized. The three-tiered model for supporting the bereaved (Killikelly et al., 2021) might help clarify the appropriate service level, who holds the correct competence to support each bereaved individual, and at what time.

### Basic Human Competence

In a project in England and Scotland, helpers were urged by the bereaved never to forget that they are human beings meeting another human being, despite their professional duties (Walter et al., 2017). Experienced compassion, respect, recognition as an individual, and kindness are essential elements for the bereaved (Cartwright et al., 2018; Walter et al., 2017). The helpers’ descriptions in our study showed an understanding that the bereaved’s grief reactions have a universal aspect, and that the bereaved’s reactions and needs are unique and individual. Additionally, the helpers described that the bereaved have lived with a “different normal”, and may feel undeserving of help due to experienced stigmatization. This comprehensive understanding of the bereaved’s situation manifested in the helpers’ practice through explaining common, normal reactions to such deaths and validating the need for help. Considering relational dialectics, this can be seen as acknowledgement, where the helpers immerse themselves in the bereaved’s world of experience while also seeing the situation from their own perspective. The helpers expressed that they had to be somewhat insistent when explaining normal reactions to sudden death. Balancing listening and sensitivity, but sometimes being more assertive based on professional knowledge and competence, demonstrates the critical interplay of comprehensive professional competence. Helpers need theoretical knowledge (about grief and drug-related death), professional-specific skills (in conducting challenging conversations), and personal competence (to emotionally engage with the bereaved). These three dimensions of competence depend on each other and work together, and ensuring that professional helpers maintain all dimensions, including personal competence, is conseqently essential.

Across all themes, the professional helpers’ respect and consideration for the bereaved individuals were apparent. From a competence perspective, this could be linked to what Skau (2017) refers to as personal competence, which includes how professionals conduct themselves as human beings and how the other person is considered in this interaction (Skau, 2017). Research shows that the qualities included in this dimension of competence can serve to counterbalance the burden of stigma, and that friendly and appropriate assistance can be health-promoting (Kalsås et al., 2022; Templeton et al., 2016). The ability to make professional discretionary assessments and considerations appears to be an essential part of the helpers’ work, and this is conveyed through numerous descriptions of how they try to sustain the unique perspectives of the various bereaved persons.

It is salient that personal competence is considered an important dimension of competence when supporting bereaved persons following a DRD. Personal competence appears to be essential for the other two dimensions, theoretical knowledge and occupation-specific skills, to come into play, as personal competence and confidence seem necessary to obtain a position to help.

### A Balancing Act Between Different Considerations

Skau’s list of characteristics constituting personal competence includes respect for oneself and others, wisdom, and the ability to act with sensitivity (Skau, 2017). The theme “A balancing act between different considerations” includes helpers’ descriptions of how they try to understand and respect the perspectives and wishes of different bereaved individuals, even if they are partly conflicting or must be weighed against legal guidelines such as confidentiality. However, the theme also includes an element of helpers’ respect for themselves by acknowledging their genuine grief over the deceased. The balancing act involves understanding, acknowledging and approaching the other’s grief while feeling one’s own grief without letting it hinder professional duties. This can be viewed through the lens of relational dialectics and the concepts of self-delineation and self-reflexivity, emphasizing the importance of emotionally understanding “the other.”

The descriptions of helpers’ genuine grief highlight that helpers are also affected by DRD. Taking this perspective seriously is essential for the care of each helper, and because the helpers’ experience, through reflection and supervision, can provide themselves and colleagues with valuable knowledge and competence for future encounters with the bereaved.

The balancing act described by helpers also involves deciding whether to allow the bereaved to maintain their autonomy and decide for themselves, or if helpers should take more leadership to ensure that the bereaved receive and accept help. Although bereaved individuals, due to DRDs and sudden deaths, generally express a desire to be respected and listened to, bereaved by sudden deaths have also expressed a desire for helpers to take the lead when needed (cf. Dyregrov, 2019, p. 109).

Balancing different considerations involves both legal and moral assessments. Many issues lack clear answers about what is legally permissible, requiring moral judgments to uphold the wishes and integrity of the various bereaved and the deceased. Such moral assessments relate to the concept of phronetic competence, also understood as practical wisdom (Hovdenak & Wiese, 2017). Many advocate for a greater emphasis on phronetic competence in professional practice. This type of competence is developed through experience and reflection (Hovdenak & Wiese, 2017; Øverlid, 2009) and should be considered part of personal competence. Øverlid (2009) argues that phronetic competence is less prioritized than theoretical competence and more practical professional skills, but is significant for health and social care professional education. Again, the importance of the personal competence dimension is highlighted, in line with participants’ descriptions of the moral considerations they make in encounters with the bereaved after DRDs.

A perspective not highlighted in the focus group interviews is the potential challenges within families and social networks around the deceased, where individual reactions and understandings may vary following drug-related death. Recognizing that each person is an individual but also in relation to others (cf. Schibbye, 2009), it is important that the support system understands and holds the necessary compentence to address these dynamics from a relational perspective.

### Strengths and Weaknesses of the Study

The strength of this study is that it is based on a large amount of data, with rich, diverse and experiential-based descriptions from a perspective of which there is currently little knowledge. The described experience came from participants with different professional backgrounds, as well as different services and municipalities. After a thorough analysis, the data yielded three solid themes that shed light on the research question.

Participants were recruited from Norwegian municipalities that had focused explicitly on overdose deaths. A more random sample from services and municipalities might have provided other descriptions of professional knowledge and awareness considering bereaved persons following a DRD. At the same time, the study called for experience-based descriptions, and it was therefore crucial that the helpers had encounters with bereaved persons following DRDs in their daily work. Several of the helpers worked in substance use services, and their experience with bereaved persons, especially persons with lives dominated by problematic substance use, was highly visible in the data. If the data collection had consisted of separate interviews with substance use services and mental health services respectively, this would possibly have brought out other nuances in the descriptions.

### Implications for Clinical Practice and Research

Defining bereaved by DRD as a distinctive group of bereaved persons may be of importance, as this may direct attention to how municipal health and welfare services can provide competent and appropriate support to this group. It seems particularly important to ensure greater competence on grief, sudden death, DRD bereavement and substance use. Additionally, a focus on further development of helpers’ personal competence and interdisciplinary collaboration for those supporting the bereaved persons seems essential.

Bereaved persons following a DRD may have complicated, complex and long-lasting strains and needs. Such an understanding and perspective on the bereaved person’s life situation may have implications for clinical practice as municipal services can adopt tools and working methods for coordination of support and care services already established (cf. Health and Care Services Act, 2011, section 3-4; cf. Norwegian Directorate of Health, 2019). The three-tiered approach could offer a valuable framework in facilitating appropriate care and fostering the necessary expertise tailored to the unique needs of bereaved individuals.

Municipal services in Norway have a great responsibility to support and care for their citizens, and many bereaved persons will probably receive the help they need from the expertise and services available at this level. However, some may need referrals to other services, and general practitioners and others who are authorised to make referrals to specialist health services will be essential in this regard. Gaining knowledge of these professional groups’ understanding and competence related to the support of bereaved persons following a DRD might be a subject for further research, and it could be of importance in ensuring that bereaved persons receive the help they need, at the right level and at the right time.

While the study is conducted within the context of Norway, the descriptions of essential competencies for effectively supporting individuals bereaved after DRDs can be considered relevant internationally, despite potential variations in organizational and public service structures.

## Closing Comments

The complex issues and the vulnerability of bereaved persons following a DRD require that helpers who support them have a comprehensive professional competence that includes distinct theoretical knowledge, occupation-specific skills and experience, and personal competence.

To support bereaved persons after DRDs appropriately, it appears to be a question of breadth and diversity in helpers’ competence, and a need for interventions across levels of services.
